# WVM-UNet: A Wavelet–Vision Mamba Framework for Enhanced Medical Image Segmentation

**DOI:** 10.3390/jimaging12080375

**Published:** 2026-08-11

**Authors:** Yulong Yang, Wen Gao, Zhengguo Wu, Chuanghua Yang

**Affiliations:** School of Physics and Telecommunication Engineering, Shaanxi University of Technology, Hanzhong 723000, China; yangyulong@snut.edu.cn (Y.Y.); gaowen@snut.edu.cn (W.G.); wuzhengguo@snut.edu.cn (Z.W.)

**Keywords:** medical image segmentation, U-Net, Mamba, State Space Models, wavelet transform, attention mechanism

## Abstract

Accurate segmentation of skin lesions and gastrointestinal polyps is essential for early diagnosis and treatment planning. Currently, Convolutional Neural Networks (CNNs) are limited by local receptive fields, missing small lesions. While Transformers model global context, their quadratic computational complexity incurs high costs. To address these limitations, we propose the Wavelet–Vision Mamba UNet (WVM-UNet), integrating State Space Models (SSMs) for linear-complexity long-range dependencies and wavelet transforms for fine-grained feature extraction. The network employs a Wavelet-based Residual State Space (WRSS) block, combining the multi-scale decomposition of discrete wavelet transforms with Vision Mamba to efficiently capture global features. A Fused Channel–Spatial Attention (FCSA) mechanism is incorporated to adaptively recalibrate feature representations. Additionally, we construct an Encoder–Decoder Semantic Connection (EDSC) to replace traditional skip connections, effectively bridging the semantic gap between cross-level features. Experimental results on multiple public datasets demonstrate the competitive performance of our method. Specifically, on the ISIC 2017 dataset, WVM-UNet achieves an mIoU of 82.94% and a DSC of 90.67%, outperforming the Mamba-based VM-UNet by 2.71% in mIoU. These results indicate our architecture effectively captures discriminative features for precise medical image segmentation.

## 1. Introduction

Medical image segmentation is a fundamental technology for computer-aided diagnosis and clinical decision-making, with its accuracy critically influencing lesion screening, surgical planning, and treatment evaluation [1]. Significant research efforts have been directed towards this area, yielding notable improvements [2,3,4]. However, deep learning-based models still face technical challenges. Convolutional Neural Networks (CNNs), exemplified by U-Net [5], are limited by local receptive fields. This restriction hinders the capture of long-range anatomical correlations, often resulting in blurred segmentation boundaries or the missed detection of small lesions [6,7].

Transformer-based models, such as TransUNet [8] and Swin-UNet [9], leverage global contextual information to enhance segmentation performance for large-scale targets. Despite their advantages, the quadratic computational complexity $O\left(N^{2}\right)$ (where N is the spatial sequence length) of self-attention mechanisms creates high computational costs when processing high-resolution medical images [10,11]. Additionally, pixel window partitioning strategies often lead to the loss of fine-grained details [12]. Consequently, developing an architecture that effectively models long-range dependencies while maintaining linear computational complexity is vital for advancing the clinical application of these technologies.

Recent advancements in State Space Models (SSMs) [13] have emerged as a promising direction in computer vision due to their efficient long-sequence modeling capabilities [14,15]. Among these, Mamba [16] offers context-aware inference by dynamically adjusting state update parameters. This model maintains linear scalability regarding sequence length, offering significant efficiency advantages over traditional self-attention mechanisms in dense data settings. In adapting SSMs for visual tasks, Vision Mamba [17] simplifies the attention mechanism to reduce memory overhead. Subsequently, medical image segmentation models such as U-Mamba [18], SegMamba [19], and VM-UNet [20] have been developed, along with VMamba [21], which integrates the Cross-Scan Module (CSM) into visual state space modeling. These methods validate the utility of Mamba-based models in balancing local details and global correlations. Nonetheless, the existing research reveals notable limitations: their unidimensional structure tends to compromise local pixel continuity in visual tasks, resulting in the underutilization of spatial modeling potential. To address these issues, this paper proposes a U-shaped segmentation model based on Wavelet-enhanced Vision Mamba (WVM-UNet). The main contributions of this work are as follows:

- To address the limitations of insufficient frequency-domain information utilization and the imbalance between global and local features in traditional models, we propose a novel Wavelet-based Residual State Space (WRSS) block. This block leverages the complementary nature of frequency-domain and spatial-domain features by integrating wavelet transform’s frequency-domain separation capability with the linear long-range dependency strengths of Vision Mamba, thereby enabling more effective feature representation.
- We design the Fused Channel–Spatial Attention (FCSA) mechanism. As a sub-module of WRSS, it dynamically adjusts the weights of dual-branch features to highlight critical information by simultaneously modeling global interdependencies between channels and local spatial details, thereby significantly improving the model’s ability to identify blurred anatomical boundaries and its overall segmentation performance.
- An Encoder–Decoder Semantic Connection (EDSC) mechanism is constructed to replace conventional simplistic skip connections. This mechanism facilitates the complementary enhancement of high-level semantic consistency and low-level detail integrity, effectively bridging the cross-level semantic gap and boosting segmentation accuracy for ambiguous boundaries and small lesions.
- Extensive experiments were conducted on public datasets covering skin lesion and polyp segmentation tasks. The quantitative results and ablation studies verify the feasibility and effectiveness of the proposed framework.

## 2. Related Work

### 2.1. SSM-Based Models

Models based on State Space Models (SSMs) originate from linear control theory and have traditionally been employed for sequential data modeling [22]. Recently, inspired by the success of Mamba in language tasks, this architecture has shown competitive performance in visual processing. For instance, VMamba introduces a framework that integrates visual patch embeddings with learnable positional information. By adopting the spatial traversal strategy from 2D-Selective-Scan (SS2D) [23], it maps feature maps into a 2D state space, mitigating the spatial correlation loss caused by 1D serialization. This allows for more accurate capture of continuous structural relationships in complex regions, such as lesion boundaries. Similarly, Vision Mamba (Vim) employs bidirectional SSMs for data-dependent global context modeling. This design overcomes the limitations of unidirectional modeling and the lack of positional awareness in the original Mamba. Moreover, Vim maintains the linear computational complexity $O\left(N\right)$ of SSMs, avoiding the quadratic $O\left(N^{2}\right)$ overhead inherent in Transformer-based self-attention.

In medical image segmentation, Mamba-based methods have been integrated into encoder–decoder frameworks [24]. Models such as U-Mamba, SegMamba, and VM-UNet utilize hierarchical state-space designs to capture global anatomical correlations while refining fine-grained edge details. Despite their efficiency, these unidimensional structures tend to underutilize spatial modeling potential and lack explicit frequency-domain priors, which are crucial for delineating complex lesion boundaries. This specific gap highlights the necessity for architectures that can simultaneously process long-range spatial dependencies and structural frequency details.

### 2.2. Wavelet-Based Deep Learning Models

The Wavelet Transform decomposes signals using basis functions with variable scales and positions, enabling the simultaneous capture of both time- and frequency-domain information [25]. This dual-domain characteristic makes it particularly well-suited for medical image segmentation compared to other spectral methods such as the Discrete Fourier Transform (DFT), Discrete Cosine Transform (DCT), and Discrete Fractional Fourier Transform (DFrFT). While DFT and DCT effectively extract global frequency components [26], they map the signal entirely to the frequency domain, thereby inherently discarding spatial localization. This is detrimental for dense prediction tasks where precise positional information of lesion boundaries is strictly required. Furthermore, although DFrFT provides a mixed spatial-frequency representation, its continuous fractional nature and complex matrix formulations incur significant computational overhead when integrated into hierarchical spatial networks [27]. In contrast, DWT seamlessly decomposes feature maps into explicit, geometrically aligned sub-bands while proportionally preserving spatial dimensions.

Building on this intrinsic property, wavelet-integrated convolution techniques have been developed to expand the receptive fields of Convolutional Neural Networks (CNNs) while maintaining computational efficiency. Consequently, these methods achieve multi-frequency response capabilities without incurring excessive parameter overhead [28]. In the realm of medical image processing, Wavelet Transform has been empirically validated to effectively enhance lesion boundary features, suppress imaging noise, and accurately preserve fine-grained anatomical details. Specifically, by decomposing images into distinct frequency sub-bands, this technique selectively enhances high-frequency components that encode critical diagnostic information—such as lesion margins and micro-vascular structures—while preserving the integrity of global anatomical structures like organ contours [29].

Recently, the integration of wavelet transforms with deep learning architectures has emerged as a prominent research direction. Its fusion with Mamba-based models has demonstrated significant potential for efficient long-range dependency modeling. However, a clear gap remains in how these diverse feature representations are integrated. Current hybrid segmentation networks typically stack spatial and frequency modules sequentially or apply standard attention mechanisms uniformly to simple concatenated features. They lack a specific mechanism to process heterogeneous spatial and frequency features according to their distinct properties, which is the key limitation our proposed model addresses.

### 2.3. Differences from Closely Related Models

To explicitly clarify the technical novelty of WVM-UNet, Table 1 provides a module-level comparison with closely related state-of-the-art architectures.

Although WVM-UNet builds upon the success of state-space models and wavelet transforms, its core components introduce distinct architectural designs compared to existing frameworks. Models such as VM-UNet and U-Mamba rely on spatial Visual State Space blocks for feature extraction. To concurrently capture spatial long-range dependencies and multi-scale frequency structural components, our WRSS module is designed with two parallel branches comprising state-space spatial modeling and discrete wavelet transform frequency decomposition. To integrate these dual-branch features, we propose the FCSA (Fused Channel–Spatial Attention) mechanism. Driven by the distinct characteristics of the inputs, FCSA applies a Channel Attention sub-module to recalibrate the state-space feature and a Spatial Attention sub-module to refine the wavelet feature. These recalibrated features are then integrated to synergize spatial and frequency information.

Furthermore, WVM-UNet adopts a specific approach to bridge the semantic gap in cross-level features. Contemporary efficient models like VM-UNetV2 employ SDI modules for hierarchical feature calibration across multiple encoder and decoder levels. Drawing inspiration from SDI, our EDSC (Encoder–Decoder Semantic Connection) mechanism focuses on dynamically balancing features at the corresponding stage rather than across all levels. It introduces a semantic matching gate configured as a learnable scalar parameter to weight the contributions of the encoder semantic feature and the upsampled decoder feature. This stage-wise alignment ensures pixel-wise consistency before fusion and effectively reduces the spatial ambiguity caused by upsampling.

## 3. Methods

### 3.1. Discrete Wavelet Transform Module

Building on the theoretical advantages discussed in Section 2.2, this section details the implementation of the Wavelet Transform module within our framework. Specifically, we adopt the Haar wavelet for its compact support and efficient representation of piecewise continuous anatomical structures. Its decomposition relies on low-pass *L*) and high-pass (*H*) filters:

(1) $$L=\frac{1}{\sqrt{2}}\left[\begin{array}{c} 1\\ 1 \end{array}\right],H=\frac{1}{\sqrt{2}}\left[\begin{array}{c} 1\\ -1 \end{array}\right]$$

**Figure 1:**
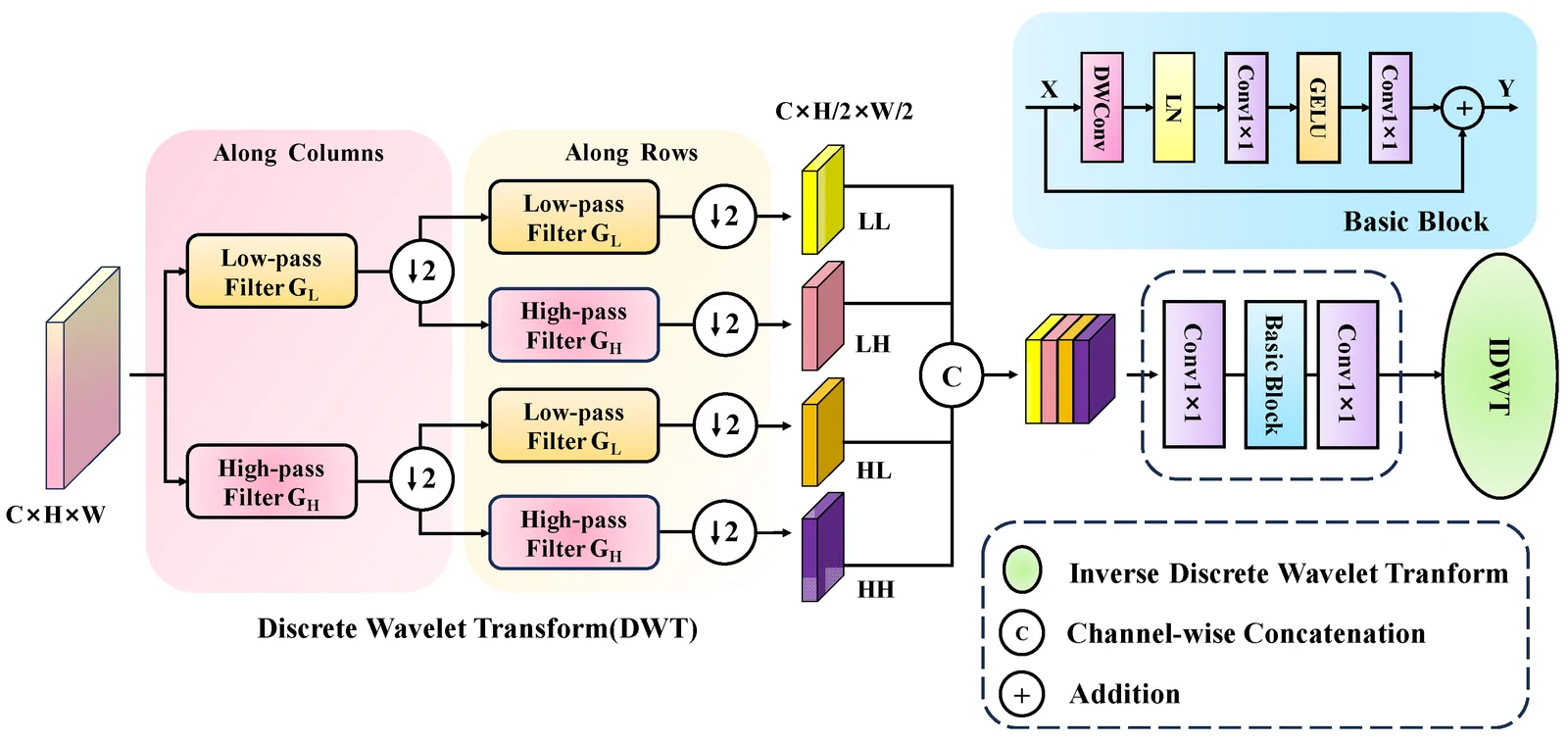
Schematic illustration of the Discrete Wavelet Transform (DWT) module. The flowchart depicts the decomposition process using low-pass and high-pass filters, the internal structure of the Basic Block, and the Inverse Discrete Wavelet Transform (IDWT).

As illustrated in Figure 1, for the input medical image feature map $F_{in}\in R^{H\times W\times C}$, the 2D-DWT decomposes the input feature map into four sub-band components with halved dimensions via two-step operations of row–column convolution and matrix transposition, which is mathematically expressed as follows:

(2) $$LL,LH,HL,HH=2D-DWT(F_{in})$$

where LL captures low-frequency structural information, and $\left\{LH,HL,\left.HH\right\}\right.$ encode high-frequency details (vertical, horizontal, diagonal). A key advantage is that Haar wavelet decomposition is information-lossless due to its bi-orthogonality property, providing a complete feature basis for subsequent enhancement. The decomposed features are reconstructed to the original scale via the 2D Inverse Discrete Wavelet Transform:

(3) $$F_{out}=2D-IWT(LL,LH,HL,HH)$$

This frequency-domain decomposition underpins the dual-branch design of our WRSS module, enabling multi-scale feature enhancement for precise lesion segmentation.

### 3.2. Wavelet–Vision Mamba UNet (WVM-UNet)

**Figure 2:**
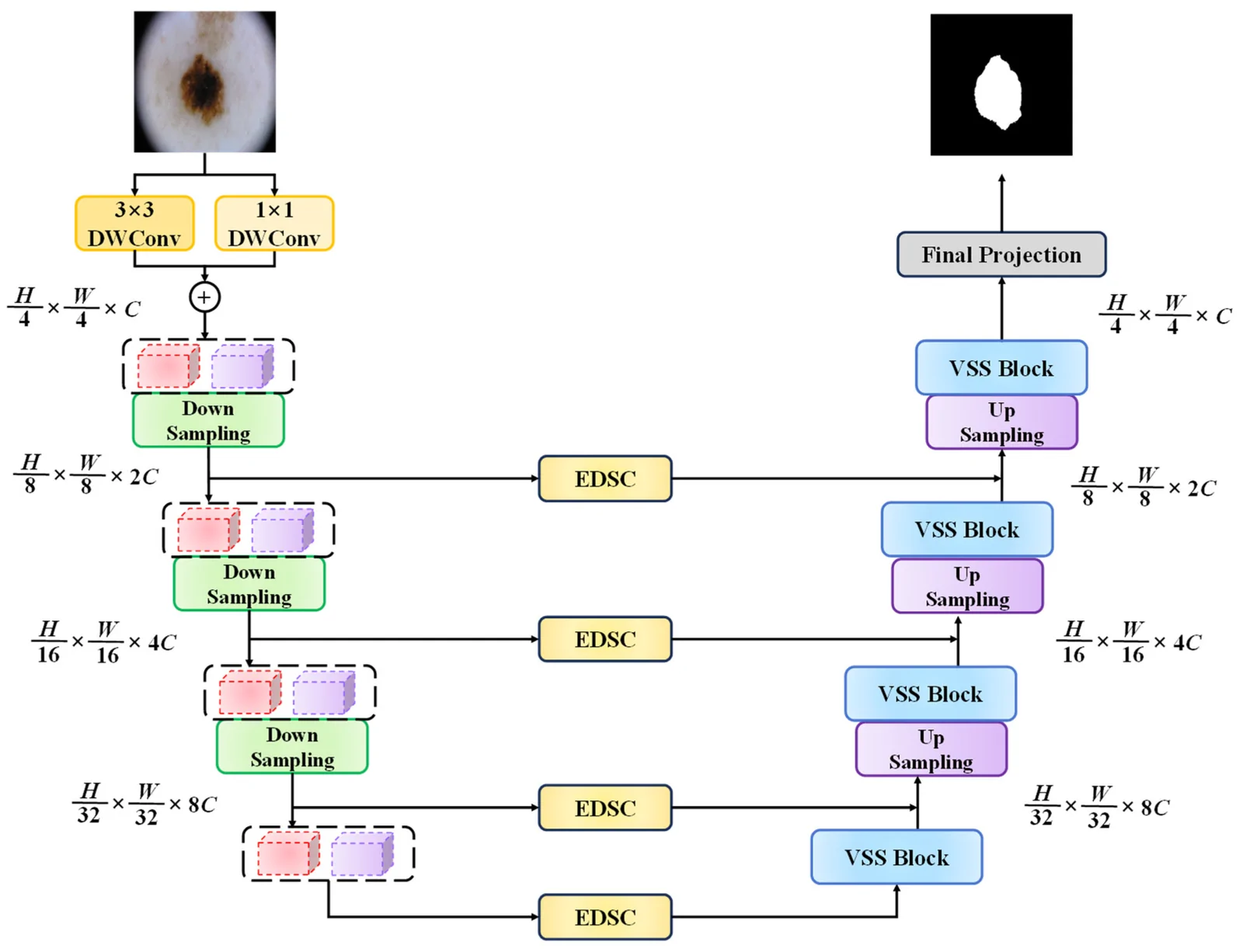
Overview of the proposed WVM-UNet architecture. The left branch illustrates the 4-stage encoder extracting multi-scale features through WRSS blocks and patch merging, expanding channels from C to 8C. The central EDSC modules align these features to bridge semantic gaps. The right branch depicts the decoder reconstructing spatial resolution via patch expanding and VSS blocks, concluding with a Final Projection layer for the segmentation mask.

The architecture of WVM-UNet is illustrated in Figure 2. Following the hierarchical structure of U-Net, WVM-UNet incorporates Wavelet Residual State-Space (WRSS) blocks into the encoder for feature extraction. Each WRSS block consists of a residual state-space modeling unit, a discrete wavelet transform module, and a Fused Channel–Spatial Attention (FCSA) mechanism. Given an input image $I\in R^{H\times W\times 3}$, the stem network, which employs parallel 3 × 3 and 1 × 1 depth-wise convolutions, maps the input to a base feature map $F_{0}\in R^{\frac{H}{4}\times \frac{W}{4}\times C}$ with an initial channel dimension of C=96.

The encoder is organized into four stages, where each stage contains WRSS blocks followed by a downsampling layer. Within the network, Layer Normalization (LN) is employed as the normalization strategy, and SiLU is utilized as the activation function. Specifically, the output feature map of the $i_{th}(1\leq i\leq 4)$ encoder stage is denoted by $F_{\mathrm{enc}}^{i}\in R^{\frac{H}{2^{i+1}}\times \frac{W}{2^{i+1}}\times 2^{i-1}C}$. To provide a clear feature hierarchy corresponding to Figure 2, the spatial dimensions are progressively halved from $\frac{H}{4}\times \frac{W}{4}$ to $\frac{H}{32}\times \frac{W}{32}$. The block depths are configured as $\left[N_{1},N_{2},N_{3},N_{4}]=[2,2,9,2\right]$, with channel dimensions doubling stage-by-stage as [C,2C,4C,8C]. The spatial downsampling operations are implemented using patch merging. Through the FCSA mechanism, WRSS blocks are designed to model both long-range dependencies and local spatial details. The decoder adopts an asymmetric 4-stage structure. To reduce computational overhead, lightweight Visual State-Space (VSS) blocks are used in the decoder instead of WRSS blocks, with block depths explicitly set to [2,2,2,1]. These blocks are paired with upsampling operations via Patch Expanding to reconstruct spatial information and progressively halve the channel dimensions. Furthermore, standard skip connections are replaced by the Encoder–Decoder Semantic Connection (EDSC) mechanism. Unlike conventional one-to-one concatenation, EDSC performs feature mapping to align encoder features with the decoder’s requirements, reducing semantic disparity between the two paths. Finally, the high-resolution features are processed through the Final Projection layer to generate the pixel-level segmentation mask.

### 3.3. WRSS Module with Embedded FCSA Mechanism

As the core feature extraction component of WVM-UNet, the WRSS module combines wavelet-based multi-scale frequency decomposition with the long-range dependency modeling of state-space units. To clearly articulate its internal structure, the detailed workflow is illustrated in Figure 2 and formally defined step-by-step. Given the input feature $F_{in}$, it is first normalized by LayerNorm to stabilize the representation, denoted by $X_{norm}=LayerNorm\left(F_{in}\right)$. Subsequently, $X_{norm}$ is processed through two parallel branches. In the first branch, the feature is encoded via depth-wise convolution (DWConv) to capture local contexts and fed into the SS2D module for long-range spatial correlation modeling. A subsequent linear layer is applied for feature projection, generating the state-space feature $F_{ssm}$. Simultaneously, the second branch performs 2D Discrete Wavelet Transform on $X_{norm}$ to decompose the signal into low-frequency structural components and high-frequency detail components, yielding the wavelet-decomposed feature $F_{wd}=2D-DWT\left(X_{norm}\right)$.

**Figure 3:**
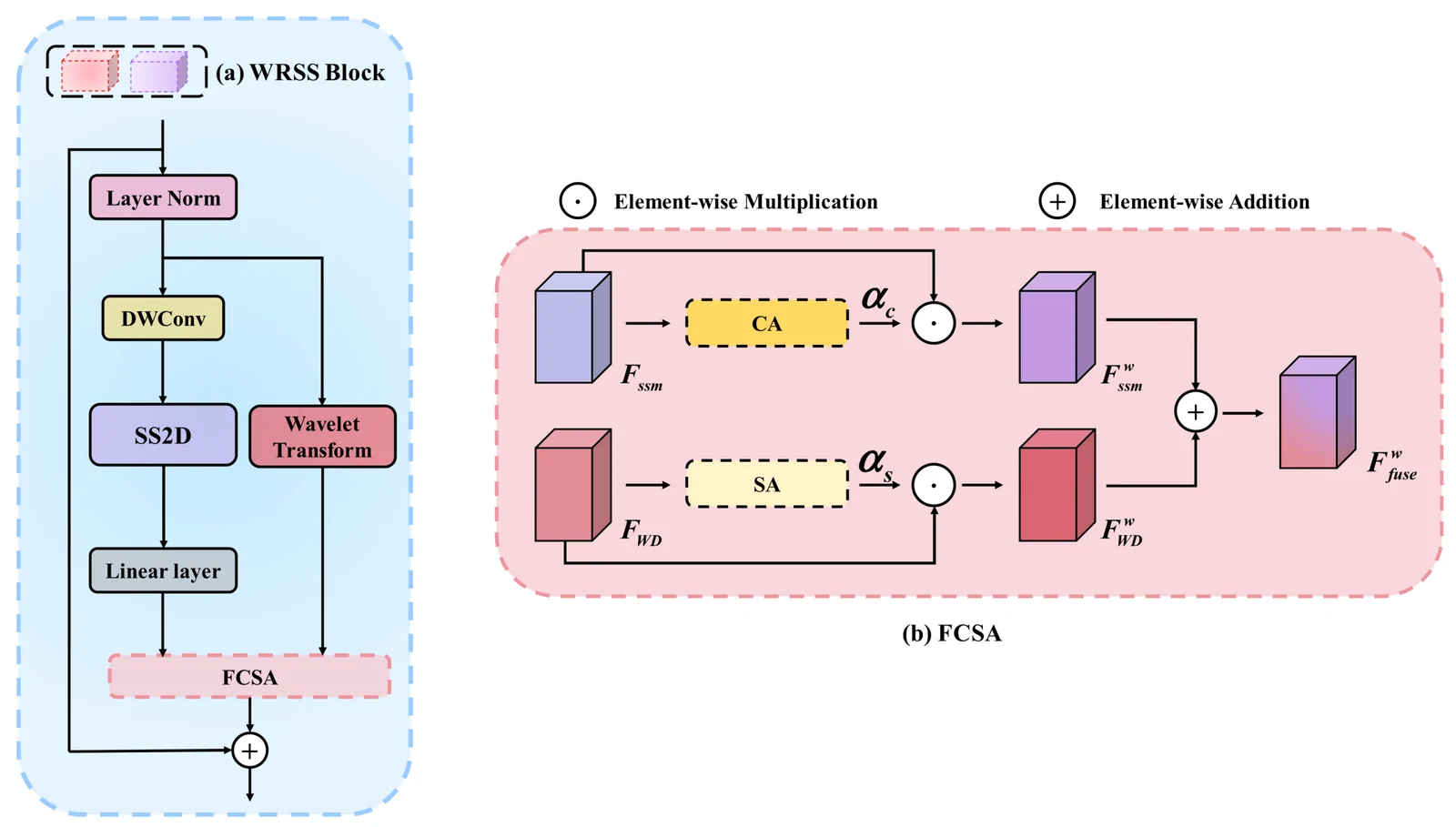
Internal components of the Fused Channel–Spatial Attention (FCSA) mechanism. It illustrates how the state-space features and wavelet-decomposed features are fused using channel and spatial attention maps.

To synergize spatial and frequency information, the Fused Channel–Spatial Attention (FCSA) mechanism is proposed, as detailed in Figure 3. The design rationale for adopting both channel and spatial attention is driven by the distinct characteristics of the dual-branch features. Furthermore, as demonstrated in previous works [31,32], the combined use of channel and spatial attention mechanisms effectively facilitates the fusion of multi-stage features of different scales, significantly improves the convergence ability of the model, and enhances its sensitivity to subtle lesions. Based on this rationale, the Channel Attention (CA) sub-module recalibrates the state-space feature $F_{ssm}$ by aggregating global context through dual-pooling, specifically Global Average Pooling (GAP) and Global Max Pooling (GMP):

(4) $$\alpha_{c}=\sigma (Conv_{1\times 1}({\bar{f}}_{c})+Conv_{1\times 1}({\bar{f}}_{c}))$$

(5) $$F_{ssm}^{w}=F_{ssm}\cdot \alpha_{c}$$

Analogously, the Spatial Attention (SA) sub-module refines the wavelet feature $F_{wd}$ by exploiting inter-spatial dependencies. To simplify, the weighted wavelet feature $F_{wd}^{w}$ is directly computed as

(6) $$\alpha_{s}=\sigma \left(Conv_{7\times 7}(cat(f_{s}^{avg},f_{s}^{\max}))\right)$$

(7) $$F_{wd}^{w}=F_{wd}\cdot \alpha_{s}$$

upon obtaining the weighted features, $F_{ssm}^{w}$ and $F_{wd}^{w}$ are fused via element-wise addition to generate the integrated representation $F_{fuse}^{w}$. To prevent the loss of original structural information during the complex dual-branch feature transformation, we introduce a residual connection that adds the initial input $F_{in}$ back to the fused features. The final output is defined as

(8) $$F_{\mathrm{WRSS}}=\mathrm{RSConn}(F_{in},F_{fuse})$$

where RSConn represents the residual connection. By integrating the FCSA mechanism, the WRSS module adaptively recalibrates dual-branch features, significantly improving the representation of fine anatomical structures and ambiguous lesion boundaries.

### 3.4. Encoder–Decoder Semantic Connection (EDSC)

**Figure 4:**
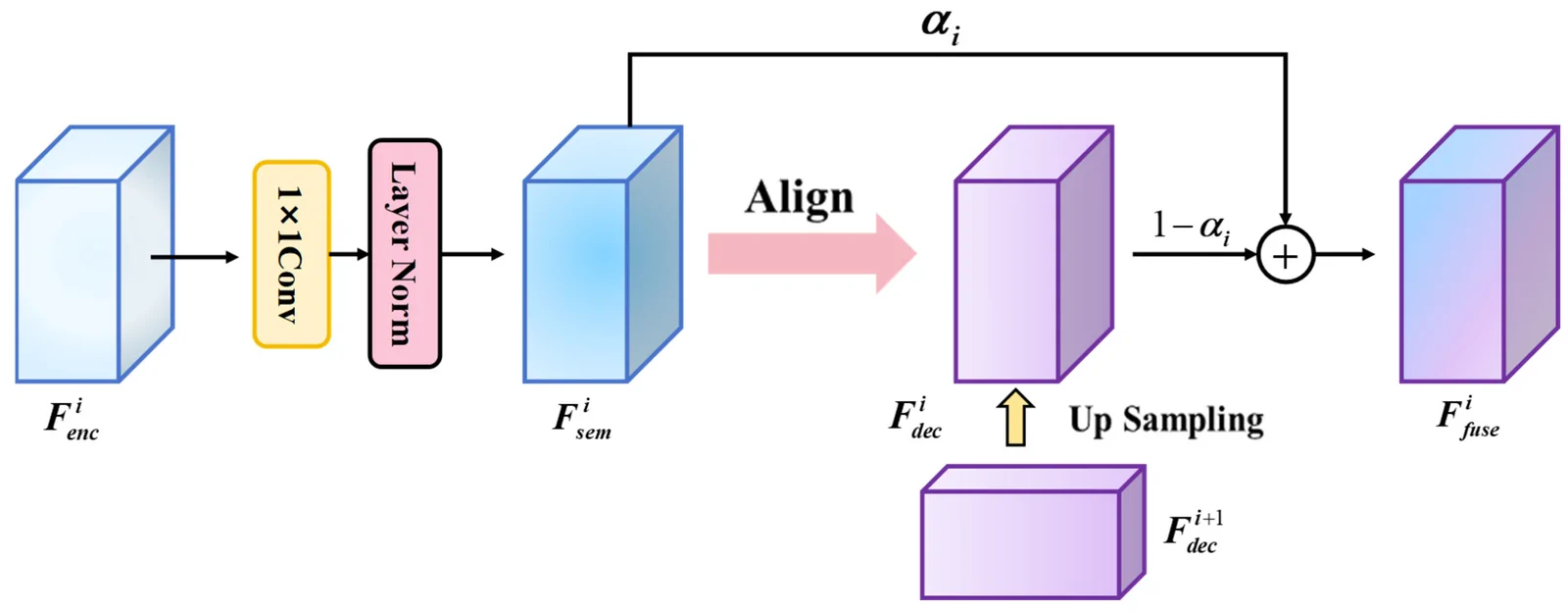
Architecture of the EDSC module, which dynamically balances encoder and decoder features using a learnable gating mechanism.

To reduce the semantic gap between cross-level features in standard skip connections, we developed the Encoder–Decoder Semantic Connection (EDSC) mechanism, drawing inspiration from the SDI modules in UNetV2 [33] and VM-UNetV2 [30]. This module uses hierarchical feature calibration and weighted fusion to align the semantics between the encoder and decoder, as shown in Figure 4.

Specifically, for the input feature $F_{\mathrm{enc}}^{i}$ from the $i^{th}$ encoder stage, we first apply a 1 × 1 convolution for channel adjustment, followed by layer norm to stabilize the distribution. This produces the encoder semantic feature $F_{sem}^{i}\in R^{H_{i}\times W_{i}\times C}$. At the same time, the feature $F_{dec}^{i+1}$ from the next decoder stage is upsampled to match the spatial resolution of $F_{\mathrm{sem}}^{i}$, yielding the preliminary decoder feature. To integrate these features, a semantic matching gate $\alpha_{i}$ is used to dynamically balance their contributions. The fused output $F_{fuse}^{i}$ is defined as

(9) $$F_{fuse}^{i}=\alpha_{i}\cdot F_{sem}^{i}+(1-\alpha_{i})\cdot Align_{i}(F_{dec}^{i},F_{sem}^{i})$$

where $\alpha_{i}\in (0,1)$ is a learnable scalar parameter constrained by a Sigmoid function. The  Align(⋅) is used to bridge the differences between the encoder and decoder. Specifically, the upsampled feature $F_{dec}^{i}$ is spatially recalibrated using a 3 × 3 convolution, guided by the high-resolution semantic information from $F_{\mathrm{sem}}^{i}$. This alignment ensures pixel-wise consistency before fusion and reduces spatial ambiguity caused by upsampling.

Unlike standard concatenation skip connections that blindly double channel dimensions and inflate parameter burdens, or dense SDI modules that introduce heavy cross-stage computational overhead, the role of EDSC is strictly defined as a lightweight, stage-wise semantic aligner. By utilizing a scalar gating parameter rather than complex attention blocks, EDSC achieves precise pixel-wise consistency while preserving the overall linear computational efficiency of the network.

### 3.5. Loss

In the medical image segmentation task proposed in this paper, we mainly adopt basic cross-entropy and Dice loss as the loss functions. Medical images frequently present significant class imbalance between small target lesions and massive background tissues. Cross-entropy provides a stable gradient descent for pixel-wise classification but is highly sensitive to background dominance. Dice loss effectively mitigates this limitation by directly assessing the spatial overlap independent of the background volume. Exhaustive evaluations in the recent literature [34] and state-of-the-art benchmark frameworks [35] firmly establish the combination of these two mechanisms as the most robust baseline for diverse medical segmentation tasks. These loss functions effectively balance the optimization objectives of pixel-wise classification accuracy and region overlap rate, and their definitions are given as follows:

(10) $$L_{B\mathrm{ce}Dice}=\lambda_{1}L_{B\mathrm{ce}}+\lambda_{2}L_{Di\mathrm{ce}}$$

(11) $$L_{Bce}=-\frac{1}{N}\sum_{i=1}^{N}\left[y_{i}\log({\hat{y}}_{i})+(1-y_{i})\log(1-{\hat{y}}_{i})\right]$$

(12) $$L_{D\mathrm{ice}}=1-\frac{2\sum_{i=1}^{N}y_{i}\hat{y_{i}}+\varepsilon }{\sum_{i=1}^{N}y_{i}+\sum_{i=1}^{N}\hat{y_{i}}+\varepsilon }$$

where N denotes the total number of samples; $y_{i}$ and ${\hat{y}}_{i}$ represent the ground-truth labels and predicted masks, respectively; ε is a smoothing factor set to $1\times 10^{-5}$ to strictly prevent division by zero; and the parameters $\lambda_{1}$, $\lambda_{2}$ are used to control the relative importance of different loss terms in the formula, and both are set to 1 by default.

## 4. Results

### 4.1. Datasets

Two types of datasets are utilized to verify the effectiveness of our framework. The first type consists of open-source skin disease datasets, including ISIC 2017 and ISIC 2018. These skin datasets are split in a ratio of 7:3 for use as the training set and test set, respectively. We set the random seed to 42 to ensure reproducible splits. Furthermore, 10 percent of the training data is internally allocated as a validation set for optimal model selection during the training phase. The second type comprises gastrointestinal polyp datasets including Kvasir SEG, ClinicDB, ColonDB, and CVC 300. To ensure a fair comparison, we strictly follow the explicitly defined experimental training settings originally established by PraNet [36]. Specifically, the training set is constructed by randomly selecting 900 images from Kvasir SEG and 550 images from ClinicDB. The testing set consists of the remaining 100 images from Kvasir SEG and 62 images from ClinicDB, as well as the entire unobserved ColonDB and CVC 300 datasets to thoroughly evaluate the generalization ability of the model.

### 4.2. Experimental Setup

All experiments were conducted on a workstation with three NVIDIA TITAN V GPUs, utilizing Python 3.9.12 and PyTorch 2.0.1 as the software environment. Input images from all datasets were resized to a resolution of 256 × 256 pixels. To curb overfitting, we incorporated data augmentation methods, specifically random flipping and random rotation. The model was trained for a total of 300 epochs with a batch size of 32. We employed the AdamW optimizer with an initial learning rate of $1\times 10^{-3}$, which was adjusted using a CosineAnnealingLR scheduler with a cycle period of 50 epochs and a minimum learning rate of $1\times 10^{-5}$. To quantitatively assess the performance, the primary evaluation metrics, including mean Intersection over Union (*mIoU*), Dice Similarity Coefficient (*DSC*), Accuracy (*ACC*), Sensitivity (*SEN*), and Specificity (*SPE*), are defined as follows:

(13) $$mI\mathrm{oU}=\frac{1}{k+1}\sum_{i=0}^{k}\frac{TP}{TP+FP+FN}$$

(14) $$DSC=\frac{2TP}{2TP+FP+FN}$$

(15) $$ACC=\frac{TP+TN}{TP+TN+FP+FN}$$

(16) $$SEN=\frac{TP}{TP+FN}$$

(17) $$SPE=\frac{TN}{TN+FP}$$

where *TP*, *TN*, *FP*, and *FN* represent the number of true positives, true negatives, false positives, and false negatives, respectively.

### 4.3. Comparison with Existing Methods

To evaluate the performance of WVM-UNet, we conducted extensive comparative experiments on two representative medical segmentation tasks: skin lesion segmentation and gastrointestinal polyp segmentation. A total of six public datasets were utilized to ensure the comprehensiveness of the evaluation. We compared our model with various state-of-the-art (SOTA) methods, including CNN-based networks like U-Net and Mamba-based architectures like VM-UNet.

The quantitative results for skin lesion segmentation are presented in Table 2. On the ISIC-2017 dataset, WVM-UNet achieves strong results across most key metrics. Specifically, our model reaches an mIoU of 82.94%, outperforming the Mamba-based baseline VM-UNet by 2.71%. While the baseline VM-UNet primarily relies on spatial state-space modeling, which struggles with complex or blurred boundaries, our 2.71% mIoU gain is primarily attributed to the WRSS block’s ability to introduce frequency-domain features via the Discrete Wavelet Transform (DWT). This explicitly allows the network to capture high-frequency edge details that are typically lost in standard SSMs, translating into higher evaluation metrics. Although VM-UNet exhibits marginally higher Sensitivity (SEN), it is crucial to recognize that models aggressively predicting positive pixels often suffer from over-segmentation, which inherently leads to increased false positives. WVM-UNet reduces this common trade-off by achieving higher Specificity (SPE) and overall Accuracy (ACC). This indicates a more favorable overall balance: our model accurately isolates true lesion regions without misclassifying the surrounding background. This competitive performance is also observed on ISIC-2018, where WVM-UNet achieves an mIoU of 81.93% and a DSC of 90.07%, showing competitive results against most competing networks.

**Figure 5:**
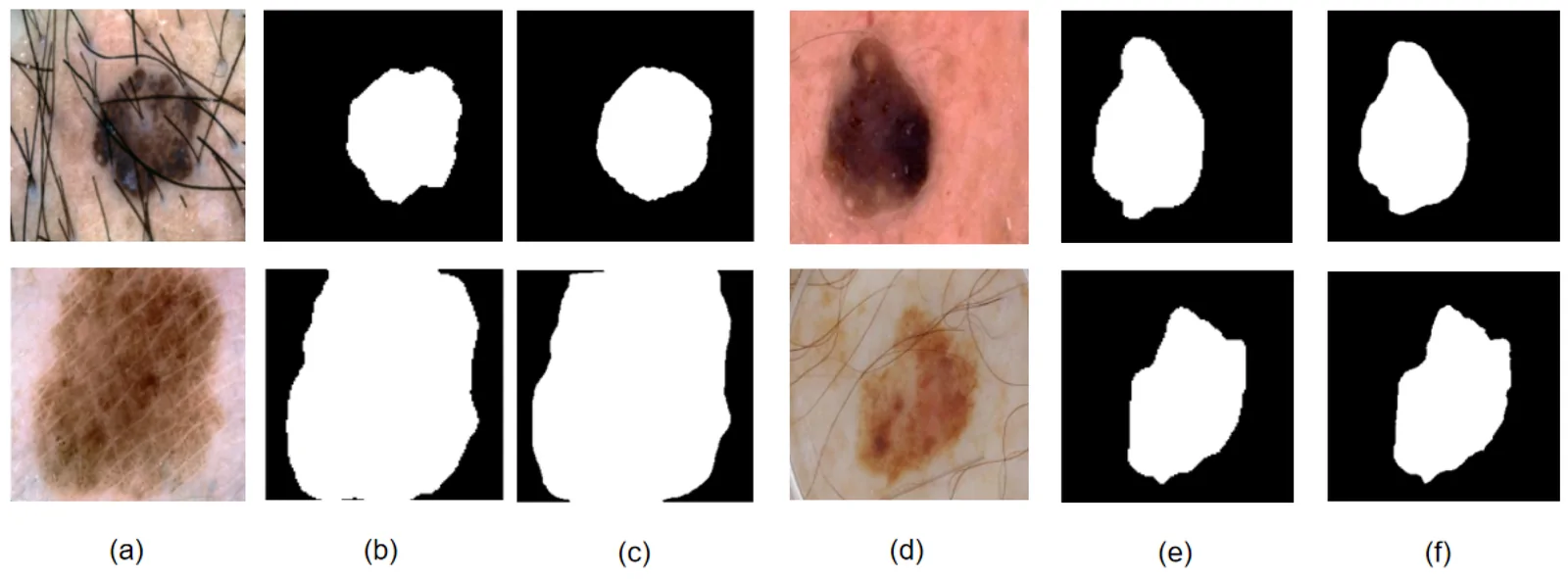
Qualitative segmentation results of the proposed WVM-UNet on challenging dermoscopy images. Columns (**a**–**c**) denote the input images, ground truth, and our predictions from the ISIC 2018 dataset, respectively. Columns (**d**–**f**) display the corresponding sequence from the ISIC 2017 dataset.

To assess the performance of the proposed architecture, Figure 5 illustrates the segmentation outputs of WVM-UNet alongside the corresponding input images and ground truth masks. As observed in column (a), dermoscopy images often contain dense hair artifacts over lesion regions. Such artifacts may mislead feature extraction networks and trigger false-positive predictions. However, our model maintains high spatial fidelity and generates smooth contours without being disturbed by these foreground occlusions. A key contributing factor behind this performance lies in the frequency-domain separation capability of the WRSS block. By integrating the Discrete Wavelet Transform (DWT), the network effectively decouples the input into distinct sub-bands. High-frequency noise (such as fine hair structures) is isolated, allowing the network to suppress these artifacts, while the low-frequency sub-band explicitly preserves the critical morphological contours of the genuine lesion.

**Figure 6:**
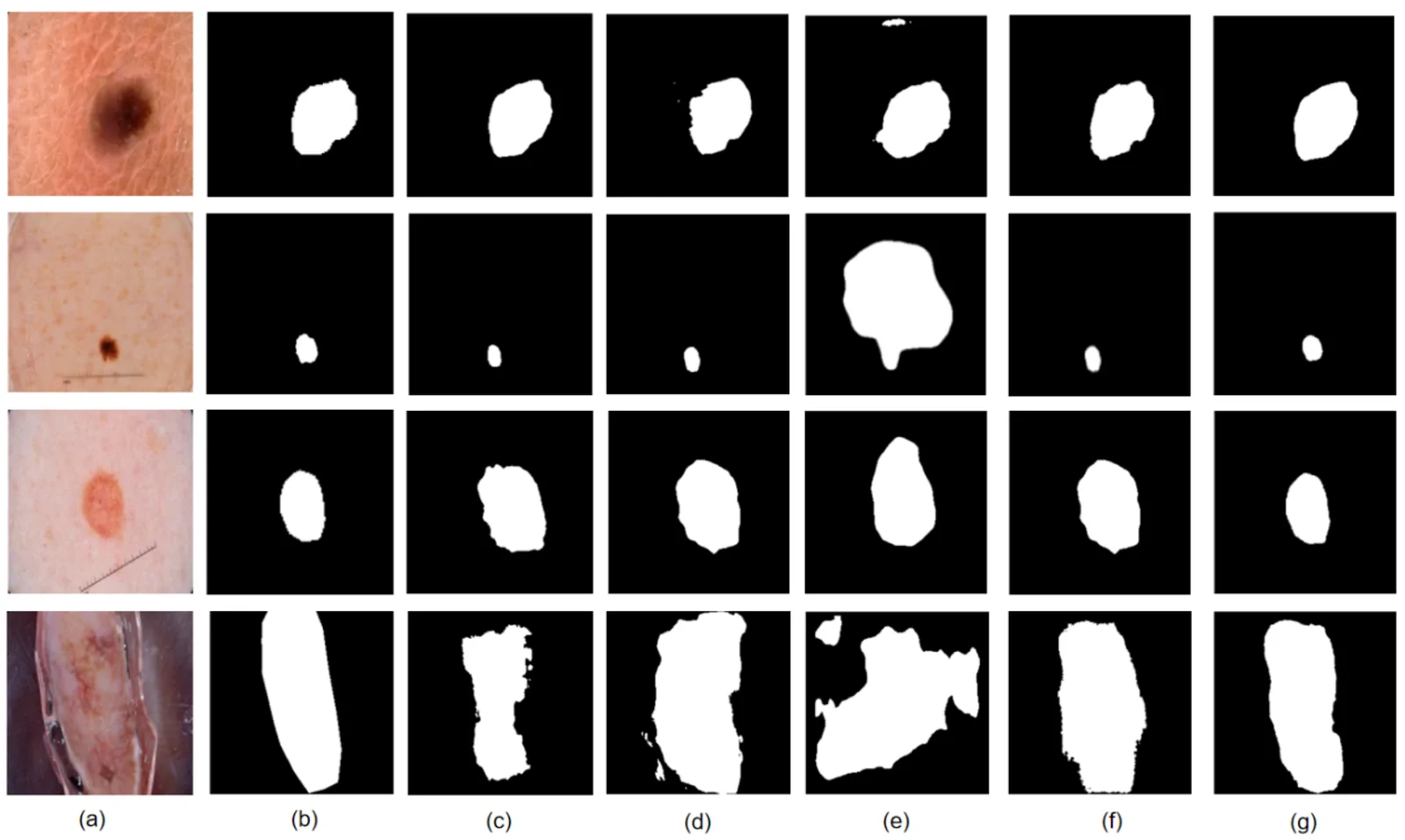
Visual comparison of segmentation results between the proposed WVM-UNet and other state-of-the-art methods on the ISIC dataset. (**a**) Input image, (**b**) Ground Truth, (**c**) TransFuse, (**d**) U-Net++, (**e**) U-Net, (**f**) VM-UNet, (**g**) Ours.

Furthermore, Figure 6 provides a comprehensive visual comparison between WVM-UNet and several representative SOTA methods across varying degrees of difficulty, including small targets, low-contrast boundaries, and complex textures. Classical CNN-based architectures, such as U-Net and U-Net++, tend to produce masks with jagged boundaries. Due to limited local receptive fields, they are prone to fragmentation or over-segmentation for large, irregular lesions. While the Mamba-based baseline VM-UNet reduces fragmentation using global receptive fields, it still shows minor boundary deviations in low-contrast areas. This is because 1D sequence serialization partly breaks 2D local pixel continuity. In contrast, WVM-UNet often generates cohesive masks with sharper, more accurate edges that align closely with the ground truth across the presented scenarios. This competitive visual performance points to two key architectural strengths: first, the EDSC module helps to bridge the semantic gap between deep and shallow layers, preventing spatial ambiguity during upsampling; second, the FCSA mechanism aids in modeling channel and spatial interdependencies, suppressing background interference while adaptively highlighting fine-grained anatomical integrity.

Table 3 highlights the generalization capability of WVM-UNet across four diverse polyp datasets. These datasets present significant challenges due to the low contrast between polyps and surrounding tissues. To provide a comprehensive evaluation against recent advancements, we incorporate state-of-the-art methods including HarDNet-MSEG, CaraNet, and Polyp-PVT and Mamba-based architectures. Notably, on the CVC-300 dataset, WVM-UNet achieves a strong mIoU of 89.98%, with an absolute improvement of 10.43% over the baseline VM-UNet. These competitive metric gains on challenging polyp datasets, which have small targets and low contrast, are attributed to the synergistic design of WVM-UNet. Instead of experiencing semantic mismatch during feature upsampling like the baseline, our EDSC module helps to mitigate the semantic gap between deep global representations and shallow local textures. Combined with the adaptive background suppression provided by the FCSA mechanism, the model effectively reduces both false negatives and false positives, resulting in competitive numerical improvements on CVC-300 and ColonDB. On the ColonDB dataset, which is known for its small and hard-to-detect targets, our model still yields a stable 6.34% gain in mIoU compared to VM-UNet. While architectures with heavy parameter counts like CaraNet and Polyp-PVT achieve marginally higher mIoU on Kvasir-SEG and ColonDB, WVM-UNet maintains highly competitive DSC scores and demonstrates robust lesion boundary delineation with a more efficient linear-complexity architecture.

It is necessary to acknowledge that the quantitative results for several comparative models are cited directly from established benchmarks. Objective performance discrepancies arise due to inherent variations in experimental configurations across different studies. Regarding data preprocessing, cited models frequently employ extensive data augmentation strategies, whereas our framework utilizes standardized spatial transformations. Furthermore, many recent architectures process higher input resolutions such as 352 × 352 pixels to secure marginal metric gains, whereas our WVM-UNet operates efficiently on a 256 × 256 pixels resolution. Differences in training schemes, such as optimizer and loss function settings, as well as dataset splitting methods across studies, inevitably lead to metric fluctuations. To mitigate these confounding factors and ensure a controlled and fair evaluation, we locally reproduced the baseline VM-UNet under the exact same hardware, dataset splits, and hyperparameters as our WVM-UNet. These results demonstrate that the linear complexity of Mamba blocks, combined with our architectural optimizations, enables the model to capture long-range dependencies more effectively than prior state-space models under these conditions.

In summary, competitive performance gains across both skin lesion and polyp datasets demonstrate the competitive segmentation accuracy and strong generalization performance of WVM-UNet. Following the deterministic evaluation protocols of our baselines we strictly fixed the random seed to guarantee reproducibility. Achieving competitive metric improvements across six independent datasets including the highly saturated ISIC-2018 benchmark demonstrates the effectiveness of our framework under fixed experimental settings. To further dissect the contribution of each core component we conduct the following ablation studies.

### 4.4. Computational Efficiency and Complexity Analysis

Beyond evaluating accuracy, we assess the computational complexity and real-world inference efficiency of different models, as depicted in Table 4. All tests are conducted on an NVIDIA TITAN V GPU with an input image size of (3, 256, 256) and a batch size of 1. To provide realistic deployment metrics, the reported GPU memory usage reflects the total allocated memory during inference, which inherently includes the necessary PyTorch CUDA context initialization overhead. Theoretically, traditional Transformer-based models suffer from a quadratic computational complexity of $O\left(N^{2}\right)$ relative to the spatial sequence length $N\left(N=H\times W\right)$, limiting their real-time application in many practical scenarios. In contrast, by leveraging the State Space Model (SSM) block, our WVM-UNet successfully maintains a linear theoretical complexity of $O\left(N\right)$.

As shown in Table 4, while U-Net and TransUNet exhibit significant theoretical computational burdens, the SSM-based VM-UNet offers a lightweight baseline. Building upon this, our proposed WVM-UNet integrates Wavelet Decomposition and Fused Attention modules while preserving the overall linear complexity. WVM-UNet refines the parameter count to 26.97 M and requires 6.91 G FLOPs. Notably, although the theoretical FLOPs slightly increase compared to the VM-UNet baseline, our architecture greatly optimizes hardware memory access and parallel computational flows. Consequently, WVM-UNet achieves a competitive inference speed of 31.08 ms and an operating speed of 32.18 FPS, while maintaining a highly competitive GPU memory footprint of 1594 MB. While U-Net achieves faster inference time and higher FPS, WVM-UNet delivers a favorable accuracy–efficiency trade-off with improved segmentation performance and fewer parameters than several competing methods, meeting typical clinical real-time deployment requirements.

### 4.5. Ablation Experiment

Ablation studies on the ISIC-2017 dataset evaluate the individual contributions of the proposed modules. As defined in Table 5, the variants integrate the Encoder–Decoder Semantic Connection (ED), Wavelet Decomposition (WD), and Fused Channel–Spatial Attention (FA). The baseline model is constructed upon a standard Vision Mamba UNet (VM-UNet) architecture. Specifically, in this baseline, feature extraction relies on basic State Space Model (SSM) blocks without frequency-domain decomposition, the FCSA mechanism is omitted, and conventional concatenation-based skip connections are used instead of the EDSC modules. We then developed four variants by progressively integrating our proposed components, culminating in the complete WVM-UNet.

From the results in Table 5, a significant fluctuation in model complexity is observed when isolating individual components. Notably, the Baseline + ED variant significantly reduces the parameter count from 27.43 M to 17.90 M while improving the mIoU to 81.80%. This substantial parameter reduction is primarily attributed to the architectural difference in cross-level feature fusion. In the baseline, conventional skip connections concatenate encoder and decoder features, doubling the channel dimension (from C to 2C) and consequently increasing the parameter burden of subsequent convolution layers. Conversely, our EDSC module employs a learnable gating mechanism for element-wise integration, maintaining a constant channel dimension (C) and effectively reducing structural redundancy. In contrast, the Baseline + WD variant increases the parameter count to 36.35 M due to the parallel network branches required for DWT decoupling. However, it is crucial to note that when all components are integrated (Baseline + ED + WD + FA), the total parameter count converges to 26.97 M, which is strictly lower than the original baseline (27.43 M). This convergence guarantees the structural fairness of our comparative experiments, demonstrating that WVM-UNet achieves performance gains through a highly efficient parameter reallocation strategy rather than simply expanding network capacity.

**Figure 7:**
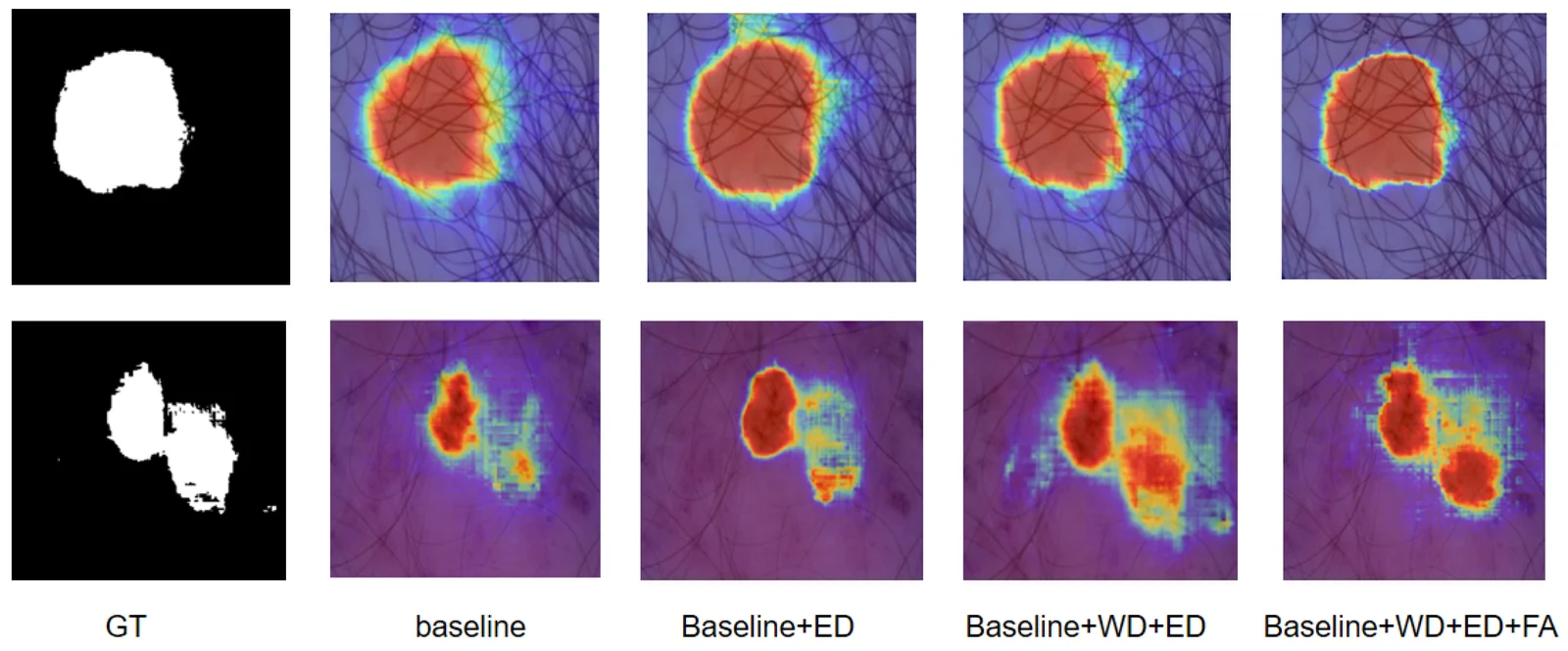
The Attention Map Overlay illustrates the progressive enhancement of the model’s attention guidance capability. Deep red and yellow regions denote high-activation areas that reflect the model’s focus on lesion regions, while blue/purple backgrounds represent low-activation non-lesion tissues.

Beyond quantitative metrics, the qualitative results in Figure 7 further validate these architectural improvements by explicitly visualizing the network’s internal focus through Attention Map Overlays. As shown in the progression, the baseline model’s attention is somewhat diffuse, frequently extending into surrounding healthy tissues and being easily distracted by background noise. With the progressive integration of the ED and WD modules, spatial fragmentation is reduced. Ultimately, in the complete architecture (Baseline + WD + ED + FA), the high-activation regions (indicated by deep red and yellow areas) become highly concentrated and closely constrained within the lesion boundaries. The structural reason behind this highly focused activation is the synergistic effect of the proposed modules: while the state-space model provides global contextual awareness, the FCSA mechanism dynamically computes channel-wise and spatial-wise weights. This adaptive recalibration guides the network to assign low weights to irrelevant background features and significantly higher weights to discriminative semantic regions, largely explaining the robust segmentation performance.

In conclusion, the ablation study demonstrates that the strong performance of WVM-UNet results from the strategic integration of semantic alignment, wavelet-based feature extraction, and adaptive attention. Each module plays a distinct yet complementary role in enhancing both the efficiency and accuracy of the model.

## 5. Discussion

The proposed WVM-UNet framework shows promising potential to support clinical workflows by providing accurate segmentation of skin lesions and gastrointestinal polyps. Clinically, precise boundary delineation is crucial: in dermatology, it directly assists dermatologists in evaluating the asymmetry and exact margins of melanomas (the ‘A’ and ‘B’ in the ABCD rule) for surgical planning; in gastroenterology, robust polyp segmentation aids endoscopists in calculating polyp volume and determining precise resection margins. Furthermore, by achieving a linear scaling law through the Wavelet-based Residual State Space (WRSS) block, the architecture maintains high computational efficiency, making it a viable candidate for future integration into resource-constrained medical hardware.

Despite the robust quantitative performance, an in-depth analysis of the model’s failure scenarios reveals specific structural limitations. Specifically, the model occasionally struggles to achieve flawless pixel-wise delineation in regions characterized by extreme low contrast or indistinct tissue transitions. The primary reason behind this limitation lies in the reliance of our WRSS block on the Discrete Wavelet Transform (DWT). The DWT inherently requires distinct gradient changes to extract high-frequency edge components. When the local tissue contrast is exceptionally low, these high-frequency signals are virtually absent. Consequently, the network is forced to depend predominantly on the global context provided by the Vision Mamba module, which, while effective for broad localization, lacks the fine-grained edge priors needed to resolve ambiguous anatomical boundaries, sometimes resulting in fuzzy margins or minor under-segmentation.

Furthermore, it is essential to acknowledge several critical limitations regarding the clinical relevance and evaluation methodology of the current study. Currently, the WVM-UNet model has only been evaluated on public, retrospective datasets. Medical imaging is inherently susceptible to dataset bias and domain shift caused by variations in hospital equipment, imaging protocols, and patient demographics. Because the model lacks validation on external hospital datasets and has not undergone prospective testing, its robustness and uncertainty in real-world clinical deployment remain open questions.

Future research will focus on two main directions to address these limitations. Architecturally, we aim to integrate edge-aware loss functions and explore adaptive frequency-feature fusion mechanisms to dynamically compensate for the loss of high-frequency edge priors, thereby bolstering boundary robustness in low-contrast scenarios. Clinically, we plan to collaborate with medical institutions to conduct rigorous multi-center external validations and prospective clinical trials, effectively bridging the gap between retrospective benchmark performance and real-world clinical utility. Additionally, all experiments in this work are conducted with a fixed random seed to ensure result reproducibility, but no multiple independent repeated experiments are performed to report the mean and standard deviation of the performance metrics. In future work, we will conduct multiple repeated experiments with different random seeds to further verify the statistical stability of the proposed model.

## 6. Conclusions

In this paper, we present WVM-UNet, a medical image segmentation framework that integrates frequency-domain enhancement with efficient long-range modeling to address the limitations of existing methods. To resolve the insufficient fusion of frequency–spatial features and the imbalance between global and local representations, we develop the Wavelet Residual State Space (WRSS) module, which synergistically combines the multi-scale decomposition of 2D Discrete Wavelet Transform (DWT) with the linear complexity long-range modeling of Vision Mamba. To sharpen blurred anatomical boundaries and highlight critical diagnostic regions, a Fused Channel–Spatial Attention (FCSA) mechanism is introduced to dynamically calibrate feature weights across dual branches. Furthermore, the Encoder–Decoder Semantic Connection (EDSC) is designed to replace traditional skip connections, effectively bridging the cross-level semantic gap and improving the segmentation of small lesions. Experimental results indicate that the synergy of these components improves precision for challenging targets while maintaining competitive computational efficiency. To address the current limitations of WVM-UNet, our future work will focus on two methodological directions. First, to improve boundary detection in extreme low-contrast regions where fixed DWT kernels struggle, we plan to investigate learnable wavelet bases and incorporate uncertainty estimation to flag highly ambiguous tissue transitions for clinician review. Second, given the linear complexity of the WRSS module, we aim to extend this 2D architecture to 3D volumetric segmentation. This will directly leverage Mamba’s efficiency to model long-range inter-slice dependencies in CT and MRI data, addressing the memory bottlenecks commonly faced by 3D Transformer models.

## Figures and Tables

**Table 1:** Module-level comparison between WVM-UNet and closely related segmentation models.

Method	Core Module	Attention Mechanism	Skip Connection Strategy	Architectural Characteristics
VM-UNet/ U-Mamba [21]	VSS Block (Spatial)	None	Standard Concatenation	Focuses exclusively on spatial state-space modeling without explicit frequency-domain decomposition.
VM-UNetV2 [30]	VSS Block (Spatial)	None	SDI	Integrates hierarchical features densely across all encoder levels, prioritizing multi-scale aggregation.
Wavelet-CNNs [3]	CNN + DWT Block	Standard Attention	Standard Concatenation	Applies attention mechanisms uniformly to fused features within standard CNN receptive fields.
Ours	WRSS Block (SSM + DWT)	FCSA (Channel Spatial-Domain Fusion)	EDSC	Integrates DWT into SSM; FCSA effectively targets heterogeneous features; EDSC aligns semantics efficiently.

**Table 2:** Comparative experimental results on the ISIC17, ISIC18 datasets (Bold indicates the best).

Method	ISIC-17				
	MIoU	DSC	ACC	SPE	SEN
U-Net [5]	76.98	86.99	95.65	97.43	86.82
UTNetv2 [37]	77.35	87.23	95.84	98.05	84.85
TransFuse [38]	79.21	88.40	96.17	97.98	87.14
MALUNet [31]	78.78	88.13	96.18	98.47	84.78
PAM-Net [39]	81.18	89.30	96.35	97.82	88.95
DEU-Net [40]	81.30	89.50	96.42	97.65	89.10
MsMANet [41]	82.05	90.10	96.80	98.35	89.10
UNetV2 [32]	82.18	90.22	96.78	**98.40**	88.71
VM-UNet [20]	80.23	89.03	96.29	97.58	**89.90**
Ours	**82.94**	**90.67**	**96.90**	98.33	89.81
**Method**	**ISIC-18**				
	**MIoU**	**DSC**	**ACC**	**SPE**	**SEN**
U-Net [5]	77.86	87.55	94.05	96.69	85.86
U-Net++ [42]	78.31	87.83	94.02	95.75	88.65
Att-Unet [43]	78.43	87.91	94.13	96.23	87.60
UTNetV2 [37]	78.97	88.25	94.32	96.48	87.60
SANet [44]	79.52	88.59	94.39	95.97	89.46
TransFuse [38]	80.63	89.27	94.66	95.74	91.28
MALUNet [31]	80.25	89.04	94.62	96.19	**89.74**
UNetV2 [32]	80.71	89.32	94.86	96.94	88.34
VM-UNet [20]	81.35	89.71	94.91	96.13	91.12
Ours	**81.93**	**90.07**	**95.19**	**96.98**	89.61

**Table 3:** Comparative experimental results on the Kvasir-SEG, ClinicDB, ColonDB, and CVC-300 datasets (Bold indicates the best).

Method	Kvasir-SEG		ClinicDB		ColonDB		CVC-300	
	MIoU	DSC	MIoU	DSC	MIoU	DSC	MIoU	DSC
VM-UNet [20]	80.32	89.09	81.95	90.08	55.28	71.20	79.55	88.61
UNet V2 [32]	84.00	91.30	83.85	91.21	57.29	72.85	82.86	90.63
VM-UNet V2 [30]	84.15	91.34	89.31	94.35	60.98	75.76	89.31	94.35
PraNet [36]	84.00	89.80	84.90	89.91	64.00	71.21	79.70	87.10
HarDNet-MSEG [45]	85.70	91.11	88.22	93.21	63.81	73.13	83.33	90.40
CaraNet [46]	**86.52**	**91.80**	88.73	93.62	68.80	77.30	83.42	90.30
Polyp-PVT [47]	86.41	91.60	88.90	93.71	**72.70**	**80.80**	83.52	90.01
Ours	84.82	91.72	**89.99**	**97.43**	61.62	76.14	**89.98**	**94.72**

**Table 4:** Comparison of computational complexity and inference time, using an NVIDIA TITAN V GPU (Bold indicates the proposed method).

Method	Input Size	Params (M)	FLOPs (G)	FPS	Inference Time (ms)	GPU Memory (MB)
U-Net	(3, 256, 256)	31.04	55.80	41.52	24.08	1518
Swin-UNet	(3, 256, 256)	27.14	11.84	28.15	35.52	1545
TransUNet	(3, 256, 256)	105.32	49.30	14.36	69.64	3120
VM-UNet	(3, 256, 256)	27.43	4.11	21.45	46.62	1562
WVM-UNet	(3, 256, 256)	26.97	6.91	32.18	31.08	1594

**Table 5:** Comparison of Ablation Experiment Results Based on the ISIC 2017 Dataset. (ED: Encoder–Decoder Semantic Connection; WD: Wavelet Decomposition; FA: Fused Channel–Spatial Attention).

Method	Params (M)	FLOPs(G)	mIoU	DSC
Baseline	27.43	4.11	80.20	89.03
Baseline + ED	17.90	4.40	81.80	90.00
Baseline + WD	36.35	6.58	81.21	89.63
Baseline + FA	27.58	4.15	80.90	89.49
Baseline + ED + WD	26.80	6.88	82.22	90.03
Baseline + ED + WD + FA	26.97	6.91	82.94	90.67

## Data Availability

The original contributions presented in this study are included in the article. Further inquiries can be directed to the corresponding author.
